# Artificial Intelligence in Breast Reconstruction: Enhancing Surgical Planning, Aesthetic Outcomes, and Patient-Centered Care

**DOI:** 10.3390/jcm14217821

**Published:** 2025-11-04

**Authors:** Brianna M. Peet, Arianna Sidoti, Robert J. Allen, Jonas A. Nelson, Francis Graziano

**Affiliations:** Department of Plastic Surgery, Memorial Sloan Kettering Cancer Center, New York, NY 10065, USA

**Keywords:** artificial intelligence, breast reconstruction, plastic surgery, machine learning

## Abstract

The integration of artificial intelligence (AI) is rapidly transforming the field of breast reconstruction, with applications spanning surgical planning, complication prediction, patient-reported outcome assessment, esthetic evaluation, and patient education. A comprehensive narrative review was performed to evaluate the integration of AI technologies in breast reconstruction, encompassing preoperative planning, intraoperative use, and postoperative care. Emerging evidence highlights AI’s growing utility across these domains. Machine learning algorithms can predict postoperative complications and patient-reported outcomes by leveraging clinical, surgical, and patient-specific factors. Neural networks provide objective assessments of breast esthetics following reconstruction, while large language models enhance patient education by guiding consultation questions and reinforcing in-clinic discussions with accessible medical information. As these tools continue to advance, their adoption in everyday practice is becoming increasingly relevant. Staying current with AI applications is essential for plastic surgeons, as AI is not only reshaping breast reconstruction today, but is also poised to become an integral component of routine clinical care.

## 1. Introduction

**Figure 1:**
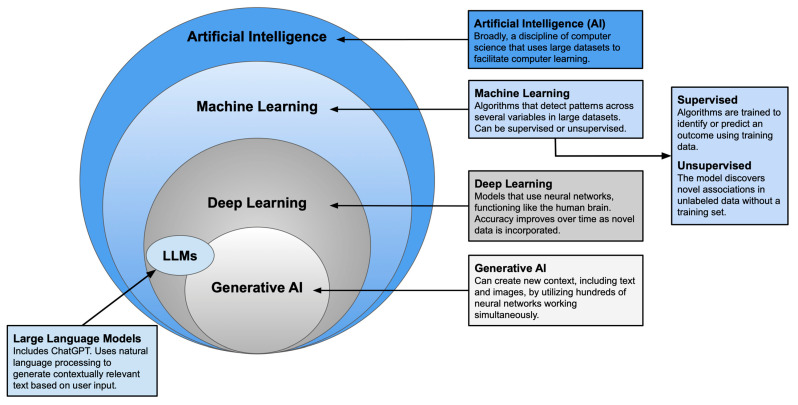
Visualizing the Relationship Between Artificial Intelligence, Machine Learning, Deep Learning.

Plastic surgery has long been defined by innovation and adaptability. As established techniques give way to newer approaches, plastic surgeons remain at the forefront of medical advancement. Artificial intelligence (AI)—an umbrella term encompassing various modes of computer learning—represents one of the most transformative developments in recent years. With tools such as machine learning, neural networks, and large language models, surgeons can now augment clinical judgment with data-driven insights to guide decision-making [1,2] (See Figure 1). AI has shown particular promise in complex procedures with specific esthetic goals [3,4], leading to its growing application in breast reconstruction.

**Figure 2:**
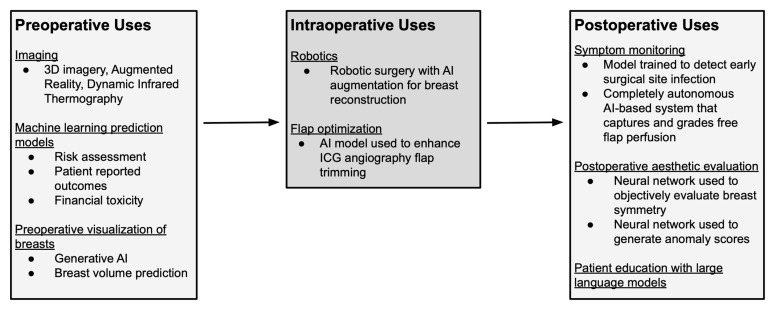
Preoperative, Intraoperative, and Postoperative Applications of AI.

Breast reconstruction is an important aspect of breast cancer care, with effects on both physical recovery and psychological well-being following mastectomy [5]. Over the past decade, AI has been incorporated into multiple stages of reconstructive care, including preoperative image assessment, risk assessment, and patient-reported outcome prediction [3,4] (See Table 1 and Figure 2). More recently, the emergence of conversational AI chatbots has prompted investigations into the accuracy and quality of AI-generated medical guidance [6]. As technological capabilities continue to expand, it is essential to examine how plastic and reconstructive surgery can evolve in parallel with these innovations.

This narrative review provides an overview of the current applications of AI in breast reconstruction, emphasizing its role in improving surgical precision, optimizing patient-centered outcomes, and identifying opportunities for future development.

Generative AI, and Large Language Models [1,2].

## 2. AI in Preoperative Surgical Planning

### 2.1. Imaging

Preoperative computed tomography angiography (CTA) imaging is the gold standard for evaluating vascular anatomy in autologous breast reconstruction. Preoperative identification of perforators in deep inferior epigastric artery perforator (DIEP) flap reconstruction enhances surgical efficiency [7]. Several technological advances now facilitate CTA interpretation and perforator mapping, with the aim to further improve operative time and outcomes [3,4].

The process of mapping vascular anatomy during presurgical planning is time consuming, requiring slice-by-slice reconstruction and manual identification of each perforating vessel by technicians and radiologists. Mavioso et al. explored whether algorithms could be used to enhance this process [8]. They compared the quality and time required for perforator identification between a traditional imaging team and an image-processing algorithm, verified against vessels found intraoperatively. The algorithm’s performance was comparable to the traditional method; it adequately identified and characterized the perforators in significantly less time, showing some benefit over operator-dependent analysis [8].

In addition to the time required to interpret the imaging, another limitation of CTA is the inability to visualize perforators in three dimensions. To address this gap, Cevik and Rosen described a novel use of generative software to render 3D images of the deep inferior epigastric artery (DIEA) and the perforators [9]. This approach offers insight into the artery’s path within a three-dimensional framework. Seth et al. expanded on this approach and used augmented reality (AR) to visualize the intra and extramuscular perforator courses of the DIEA to enhance surgical precision [10]. AR overlays digital information (like imaging) onto the user’s view of the real world, allowing for increased interactivity and immersion that is not possible with traditional imaging alone. Berger et al. evaluated an AR tool that superimposes magnetic resonance angiography (MRA) scans onto the patient in real time [11]. First, MRA images were obtained and uploaded to the augmented reality software. Then, the surgeon used the AR goggles to align the processed image data with the patient’s body. They found that the MRA AR projection closely correlated with the actual vessel location on Doppler ultrasound. Three-dimensional imagery and AR have the potential to enhance the surgeon’s understanding of individual anatomy, but the technology is in its early stages and will require additional modifications before it can be adopted on a larger scale.

Dynamic infrared thermography (DIRT) is a noninvasive technology that uses heat mapping to quickly identify perforators for reconstructive planning [12]. After establishing a baseline by cooling the abdomen, thermal imaging is used to localize hotspots correlating with areas of optimal perfusion. After two and a half minutes, these areas are marked as probable perforators. Meier et al. integrated DIRT technology with AR software to improve vessel identification in patients undergoing DIEP flap reconstruction [12]. In their study, the AR device was equipped with a thermal camera, projecting a color-coded heat map onto the patient’s abdomen in real time. They found that most DIRT-identified perforators were also identified on Doppler and CTA, and DIRT performed similarly to CTA when correlating vessels intraoperatively [12]. Although the technology is still being perfected, DIRT has the potential to optimize presurgical planning by reducing imaging time and eliminating the need for radiation and contrast exposure [12].

### 2.2. Preoperative Risk Assessment

Machine learning (ML), a subset of AI, is particularly well-suited for pattern recognition. By analyzing large datasets, ML models can independently predict outcomes with high accuracy [1]. Traditionally, risk calculators have relied on multivariate statistical models, but these tools are limited in their ability to account for the complex interplay between patient demographics, clinical and oncologic factors, and surgeon-specific choices [13]. In contrast, ML models can leverage nonlinear relationships among numerous variables to more accurately estimate complication risk. Importantly, as additional data are incorporated, these models continuously adapt and improve their predictive performance [1]. Recent studies have applied ML to predict a wide range of postoperative complications in both autologous and implant-based breast reconstruction.

In implant-based breast reconstruction, Bavaro et al. developed a model to estimate the risk of capsular contracture in patients undergoing immediate implant reconstruction followed by postoperative radiotherapy [14]. Chen et al. similarly created a model to predict capsular contracture in patients undergoing two-stage breast reconstruction [15]. ML has also been used to predict mastectomy skin flap necrosis (MSFN), with Hassan et al. identifying key risk factors and developing a model that achieved a mean predictive accuracy of 89% [16]. In addition to these studies, Chen et al. developed a machine learning model to estimate the risk of complications following tissue expander (TE) placement [13]. The model not only identified the key risk factors for TE loss, infection, and seroma, but also ranked them according to their relative contribution to each outcome. Building on these findings, the authors created a clinical prediction app using the highest-performing models, providing surgeons with a user-friendly tool to input patient-specific data and generate individualized risk scores in the preoperative setting [13].

ML models have also been applied to predict postoperative complications in the autologous breast reconstruction population. O’Neil et al. developed a machine learning model that predicted DIEP flap failure with 95% accuracy [17]. While their multivariate analysis was unable to determine predictors of flap failure, the ML model successfully recognized clinical patterns associated with increased risk, highlighting the advantages of ML over conventional statistical methods [17]. Beyond flap failure, readmission remains a significant challenge following DIEP flap reconstruction. Using the National Surgical Quality Improvement Program (NSQIP) database, Ozmen et al. created a model that predicted readmission with 89% accuracy [18]. Similarly, Gabay et al. used NSQIP data to assess short-term reoperation and readmission rates in both autologous and alloplastic breast reconstruction. Their analysis demonstrated that ML algorithms outperformed traditional logistic regression, further underscoring the utility of ML for risk prediction [19].

As ML models evolve and enter clinical practice, they hold significant potential to refine surgical risk counseling by providing personalized, data-driven outcome predictions. With continued refinement, the clinical factors included in these models—such as preoperative BMI, age, smoking status, tumor pathology, radiation details, incision length, mastectomy weight, lymph node harvest, and reconstruction type—are becoming increasingly specific. ML algorithms can synthesize these variables to identify patterns and generate individualized risk profiles. However, most current models remain preliminary and lack validation in larger cohorts. Recently, Meyer et al. demonstrated that their ML model predicting nipple-areolar complex necrosis retained its accuracy in a larger dataset [20], supporting the validity and generalizability of this approach. Looking forward, user-friendly ML calculators will likely be integrated into preoperative counseling, enhancing discussions on risk optimization and expected outcomes.

### 2.3. Patient-Reported Outcome Prediction

In addition to risk estimation, ML models can also be used to preoperatively predict patient-reported outcomes (PROs). In breast surgery, PROs are most commonly assessed with the BREAST-Q [21,22]. Using the BREAST-Q, Pfob et al. developed and validated machine learning algorithms to predict individual satisfaction with breasts at 2-year follow-up, with the goal of better informing reconstructive decision-making after cancer-related mastectomy [23]. The models were developed and validated using separate patient cohorts, and the best performing model achieved an AUC of 0.87. Notably, Pfob and colleagues were able to show that the drivers of short and long-term satisfaction with breasts were distinct, enhancing our understanding of how PROs change over time [23]. The same authors conducted a similar analysis in 2023 focused on breast satisfaction at 1 year follow-up and found 30% of women in the cohort experienced a clinically meaningful reduction in breast satisfaction [24]. They postulated that if these women had been identified using the predictive ML model, a different reconstruction option would have been recommended, and the likelihood of achieving optimal breast satisfaction would have increased substantially [24].

In addition to breast satisfaction, the BREAST-Q also assesses physical, sexual, and psychosocial well-being, providing broader insight into quality of life. A large multi-institutional study of eleven sites across North America expanded on this approach by developing and externally validating ML models to predict clinically meaningful changes in physical, sexual, and psychosocial well-being at 2 years [25]. Using data from over 1400 patients, the algorithms demonstrated acceptable predictive accuracy (AUCs 0.64–0.82 across domains), with baseline PROs again exerting the greatest influence on model performance. These findings highlight the potential of ML to forecast long-term quality-of-life outcomes and to support shared decision-making in postmastectomy breast reconstruction.

More recently, Chen et al. advanced this work by demonstrating that their ML models could predict outcomes across five distinct BREAST-Q domains [26]. The models were trained on a larger patient cohort that incorporated multiple pre-existing comorbidities. With these enhancements, the algorithms outperformed earlier models focused on breast satisfaction, physical well-being, and sexual well-being by a significant margin. These findings underscore that incorporating larger and more diverse patient datasets into ML models improves predictive accuracy and allows the algorithms to evolve over time, better capturing patient experiences.

### 2.4. Financial Toxicity

Beyond the physical appearance of breasts after reconstruction, the financial burden of cancer treatment is an important outcome to consider. Financial toxicity refers to the material, psychological, and social burden caused by treatment-related out-of-pocket healthcare costs [27]. Patients undergoing cancer therapy experience disproportionately high rates of financial toxicity, yet until recently, it was unclear which factors most strongly contributed to this burden. To address this gap, Sidey-Gibbons et al. applied ML models to study financial toxicity [28]. They found that patients receiving autologous breast reconstruction and chemotherapy faced the greatest financial strain and were more likely to adopt maladaptive coping strategies, such as skipping appointments, rationing medications, or cutting back on nonmedical spending. With this ML approach, surgeons can now identify patients at greatest risk for financial toxicity and proactively connect them with supportive resources.

## 3. Intraoperative AI Applications

### 3.1. Robotics

Robotic-assisted surgery (RAS) has revolutionized several surgical specialties, helping to minimize patient morbidity, optimize precision, and enhance intraoperative visualization [29]. Within plastic surgery, there has been a steady increase in RAS use, including in breast reconstruction, over the past decade. The main advantages of robotic systems are high-definition 3D visualization, tremor reduction, and motion scaling that enables precise micromovements. When combined with AI, these systems can further support real-time tissue identification, recognition of anatomical landmarks, and personalized surgical guidance informed by preoperative planning data [30]. The use of robotics with AI/AR augmentation in breast reconstruction remains largely conceptual; however, implementation of computer vision offers potential to enhance surgical techniques, anatomical visualization, and intraoperative navigation.

### 3.2. Flap Optimization

Indocyanine green fluorescence angiography (ICGFA), a tool that uses fluorescence to visualize tissue perfusion intraoperatively, has helped reduce perfusion-related complications after DIEP flap reconstruction [31]. Despite its utility, ICGFA is inherently subjective, making it difficult to determine the exact amount of flap debridement required to optimize surgical outcomes. Ongoing efforts have shown early success in developing high-fidelity AI models using ICGFA to assist in flap trimming decisions intraoperatively. Singaravelu et al. developed an ensemble subspace k-nearest neighbor model that was able to predict the optimal excision area with 99.3% accuracy [32]. This study offers a proof of concept that AI can help assist with intraoperative decision-making.

## 4. AI Use in Postoperative Care

### 4.1. Symptom Monitoring

In the postoperative period, prompt evaluation of potential complications is critical for optimal treatment and improved outcomes. Remote symptom monitoring has decreased the need for in-person appointments, but consequently, physicians are receiving an increasing number of postoperative wound images submitted through online portals. Reviewing these images generates significant clinician workload, indicating a need for automatization of this process. In an effort to reduce clinician burden, Muaddi et al. developed a model trained to detect surgical incisions and early surgical site infections (SSIs) [33]. The model achieved modest predictive accuracy for identifying SSIs, suggesting this tool is best suited as a triaging modality that supports a surgeon’s clinical judgment.

Similarly, frequent free flap monitoring in the early postoperative period is essential for timely detection of complications, but repeated physical exams are time-intensive and place added strain on already understaffed healthcare teams. Kim et al. created an automated AI-based flap monitoring system, using images taken with a smartphone, to reduce the need for human intervention [34]. First, they built a deep learning model capable of detecting abnormalities in flap perfusion. Then, they integrated the model into a clinically applicable system. Smartphones were installed in places where the flap could be observed and were programmed to take pictures at regular intervals. If the system detected an abnormality, medical staff was alerted directly to investigate. This system is the first to be fully automated, functioning independently without the need for additional verification of its findings. When tested in the clinical setting, the system performed reliably and smoothly. As these systems become more advanced, they will achieve more precise detection of changes in color and temperature and could potentially lead to improved flap salvage rates.

### 4.2. LLM Utilization for Patient Education

When preparing for the postoperative period, patients often need clear, reliable guidance on recovery timelines, red-flag symptoms, and drain care. In settings where immediate physician input is unavailable, investigators have examined whether large language models (LLMs) can deliver reliable medical information. LLMs—delivered as text chatbots or avatar-based videobots—can translate complex information into plain language and capture questions between visits. In 2023, Liu et al. compared ChatGPT responses to medical questions with standard Google search results, assessing for readability and reliability; five experienced breast surgeons judged ChatGPT superior in accuracy and quality, yet 40% were reluctant to endorse either platform for patient education [35]. In a separate analysis addressing breast implant–associated anaplastic large cell lymphoma and breast implant illness, the same group again found ChatGPT’s answers more comprehensible than Google’s but noted instances of inaccessible citations [36].

In 2024, Kenig et al. found that patients are comfortable with AI involvement in their care when used alongside a clinician, with trust dropping when the surgeon was absent [37]. In a study by Kim et al., patients found breast reconstruction counseling videobots to be more engaging but no more effective than chatbots for usability/accuracy, and patients still viewed both as supplements rather than replacements for surgeon counseling [6]. Collectively, these findings suggest that LLMs may serve as adjuncts to patient education, but their use should be accompanied by clinician oversight and transparent source attribution.

Early iterations of LLMs, including ChatGPT, were criticized for “citation hallucination”, i.e., the generation of fabricated or unverifiable references. This behavior reflects the probabilistic, next-token nature of LLMs rather than retrieval from curated bibliographic databases; when a specific source is not represented in the training data or retrieval context, the model may synthesize a plausible-seeming citation to satisfy the prompt [38]. Although citation handling has improved, the risk of spurious or inaccessible references persists. Nevertheless, LLMs can provide accurate, readable explanations and adjust the complexity of information to match patient health literacy. Accordingly, LLM outputs should be used as adjuncts to education—valuable for helping patients formulate questions—while all medical guidance derived from LLMs is verified by a qualified clinician.

## 5. AI in Esthetic Evaluation

### 5.1. Preoperative

Generative AI refers to technology that focuses on enhanced visualization. Crisalix is a generative AI platform that renders detailed images of the patient with a specified modification; for breast reconstruction, Crisalix transforms an image of preoperative breasts into the most likely representation of the postoperative outcome following autologous or alloplastic plastic surgery [39]. Instead of viewing “representative” postoperative images, patients can see a rendition of their own breasts after surgery. When put into practice, these visual tools will help surgeons and patients set realistic expectations for postoperative outcomes.

### 5.2. Intraoperative

A major challenge in unilateral implant-based breast reconstruction is achieving symmetry with the contralateral breast in both shape and volume. The reconstructed breast can appear fuller and more rounded following implant placement. Consequently, symmetry can be difficult to achieve in patients with small to medium-sized, ptotic breasts or limited upper pole fullness. In these cases, a contralateral balancing procedure—commonly breast augmentation—may be required to create a long-lasting, symmetric, and esthetically pleasing result. The aim is to match the contralateral breast as closely as possible. Because of this expectation, choosing the correct size implant is of importance. Chen et al. developed two automated whole breast segmentation algorithms to calculate breast volume, which could be used for breast prosthesis selection [40]. Additionally, they tested an MR-based ML model that could reliably measure breast density, a known modifiable risk factor for breast cancer. They found that the proposed segmentation algorithms were highly concordant with the manual segmentation performed by a radiologist, and importantly, they exhibited small improvements in reliability. By determining the exact breast volume preoperatively, segmentation algorithms have the potential to optimize implant selection and minimize the need for intraoperative sizers, leading to more intraoperative efficiency and patient satisfaction [40]. Breast augmentation for the contralateral breast is not without its own risks. Issues such as capsular contracture and ALCL are possible and patients should be counseled preoperatively [41].

### 5.3. Postoperative

By convention, when plastic surgeons assess postoperative reconstructive results, they use key anatomic features, including the sternal notch, nipple–areolar complex, and the inferior and lateral boundaries of the breast, as landmarks to compare breasts against each other. Although normal anatomical values exist, relying on breast features alone does not capture the subjective nature of the “esthetically ideal” breast, making accurate evaluation difficult. Neural networks (NN) are a subtype of AI that function similarly to the human brain; as more data is fed into the network, some neural connections are pruned while others are strengthened until the network can accurately predict an outcome. To help improve the quality of breast symmetry assessment, Kenig et al. designed and tested a NN capable of locating key breast features [42]. The NN was trained using a dataset of preoperative and postoperative breast images from patients undergoing cosmetic surgery and was then tested on patients undergoing breast reconstruction following oncologic surgery. Their NN demonstrated a 97.4% success rate in detecting key breast features, an impressive level of accuracy given the considerable variability in breast morphology following reconstruction and radiation therapy [42].

To evaluate differences in esthetic outcomes in patients that received postoperative radiation therapy, Kim et al. used a NN to detect anomaly scores, a measure of “loss” of features between normal training images and reconstructed images [43]. They found that autologous reconstruction showed lower anomaly scores than tissue expanders, and time elapsed since radiation treatment was correlated with significantly higher anomaly. With this data, surgeons can help patients better understand how their breasts have changed over time, and if applicable, counsel on the benefits of revision surgery for achieving the desired esthetic result.

When communicating asymmetry following breast reconstruction, care must be taken to fortify the evaluation with medically accurate data while also validating the emotional experience of breast cancer recovery. Kenig et al. reviewed symmetry evaluations conducted by LLMs like ChatGPT version 4o and Gemini 1.5 to determine if they can deliver information reliably and empathetically [44]. While ChatGPT was able to rate symmetry on a 0–10 scale, it did not modulate the delivery of its assessments, even if the score was low. LLMs are unable to detect social cues, thus they are unlikely to consider the gravity of a low score and its potential impact on the patient’s well-being and self-worth. AI and NNs excel at removing the inherent subjectivity of human esthetic evaluations. But the results of these studies make it clear that delivering sensitive information should remain in the hands of the physician.

## 6. Limitations of AI

While the use of AI in plastic surgery is rapidly expanding, important limitations remain. In this review, we have outlined several ways that AI can improve efficiency at all stages of breast reconstruction. However, the implementation of certain AI modalities, particularly AR and LLMs, on a large scale can be costly. The expense incurred by the healthcare system would have to be weighed against the quality of care provided with AI—further work exploring the cost effectiveness of AI is needed. Additionally, if an AI tool causes an adverse outcome, where does the liability fall? Universal guidelines must be established to ensure patient safety is prioritized and the boundaries of AI are well defined.

Furthermore, surgeons should be cautious in relying on AI to interpret subjective breast reconstruction outcomes. A “successful” reconstruction is multifactorial and inherently subjective, encompassing not only esthetic parameters such as symmetry, contour, and nipple position but also patient-reported satisfaction, psychosocial well-being, and restoration of body image. These dimensions are difficult to quantify objectively, and current algorithms often rely on surrogate visual metrics or limited datasets that fail to capture the nuances of patient perception. As such, AI-derived assessments should be viewed as adjunctive tools rather than definitive evaluators of reconstructive success, and their outputs must always be interpreted in the context of clinical judgment and patient experience.

Although ML models, NNs, and LLMs can adapt as they are exposed to new data, their performance is constrained by the datasets on which they were originally trained. As clinical guidelines and surgical techniques evolve, AI systems may fail to reflect these changes until updated datasets are incorporated. Similarly, the accuracy and applicability of AI tools are limited by the diversity of the training population. Certain patient experiences are inevitably underrepresented in early model development, and without careful selection of representative samples, prediction accuracy may decline when models are validated in broader or distinct patient cohorts. Additionally, heterogeneity in breast reconstruction techniques, including variation in incision patterns, implant pocket selection, use of acellular dermal matrix, and intraoperative decision-making, introduces substantial variability in postoperative outcomes. Differences in surgeon experience, institutional protocols, and reconstructive philosophy further compound this heterogeneity, making it challenging to train AI models that generalize effectively across institutions. As a result, algorithms developed in one setting may perform poorly when applied elsewhere, underscoring the need for large, multicenter datasets and standardized surgical documentation to enhance model robustness and external validity. Bias is another concern. AI systems risk perpetuating or even amplifying racial and socioeconomic disparities in healthcare. For instance, if a training dataset predominantly consists of Caucasian patients from higher socioeconomic backgrounds, the model will optimize its performance for that population, while predictions for underrepresented or marginalized groups may be inaccurate or unreliable [45]. To enable safe and equitable implementation, large-scale AI adoption will require the development of robust, diverse databases that capture the full spectrum of patient demographics and clinical experiences.

## 7. Future Directions

The integration of artificial intelligence into breast reconstruction is still in its early stages, with several priorities for advancement. Large-scale, multi-institutional validation of current models is essential to confirm accuracy across diverse populations and reconstructive approaches. Algorithms predicting outcomes such as flap failure, tissue expander loss, and capsular contracture have shown promise, but broader datasets are needed to ensure generalizability. Future models should also expand in scope. Incorporating variables such as perforator anatomy, mastectomy weight, incision type, radiation exposure, and use of acellular dermal matrix could refine complication prediction and operative planning. Embedding these systems into electronic health records or mobile applications will facilitate adoption by surgeons and empower patients during decision-making. As these tools mature, ethical considerations will remain critical. Safeguards around data privacy, algorithmic bias, and the delivery of sensitive esthetic assessments are necessary to maintain trust and equity in care. Ultimately, the greatest potential lies in integrated AI ecosystems that combine predictive modeling, esthetic evaluation, and patient education into a single pathway. Such platforms could align preoperative counseling, surgical execution, and postoperative monitoring, enhancing both outcomes and the overall patient experience after breast cancer treatment.

## 8. Conclusions

Plastic surgery has always been driven by innovation, and AI now stands at the forefront of this evolution. Surgeons are beginning to incorporate AI across multiple aspects of breast reconstruction care. Machine learning models have shown promise in risk estimation, enabling prediction of postoperative complications and patient-reported outcomes based on clinical, surgical, and patient-specific variables. These tools are increasingly accurate and are expected to become accessible as phone-based applications for use in the clinic. Neural networks can provide objective, quantifiable assessments of breast esthetics, addressing what has traditionally been a subjective outcome. Large language models further support patient care by guiding discussions during consultations and reinforcing in-clinic education with accessible medical information.

Looking ahead, AI will enable plastic surgeons to refine breast reconstruction surgical planning, enhance patient outcomes, and expand the scope of reconstructive care. Widespread adoption, however, will depend on reducing costs, improving usability, and establishing rigorous safety and ethical guidelines. While AI holds immense potential to transform reconstructive surgery, it will remain a powerful adjunct—never a replacement—for the experience, judgment, and compassion of the human surgeon.

## Figures and Tables

**Table 1:** Benefits and Challenges of AI Applications in Breast Reconstruction.

Phase	AI Application	Benefits of AI	Challenges of AI
**Preoperative**	3D imagery, augmented reality, dynamic infrared thermography	-Immersive visualization of perforators for flap reconstruction -Faster imaging without the need for radiation or contrast	-High cost -Clunkiness of augmented reality software
	Machine learning for risk assessment, patient reported outcome prediction, and financial toxicity prediction	-Has the ability to leverage nonlinear relationships among numerous variables -Can be incorporated into apps for easy-to-use in clinic predictions	-Accuracy is contingent on the training datasets, biases can sometimes be introduced
	Generative AI for preoperative visualization of breasts	-Patients can see a rendition of their own breasts after surgery -Preoperative breast volume estimation can ensure precise implant selection	-Costly and not readily available at most clinics
**Intraoperative**	AI model used to enhance ICG angiography flap trimming	-Eliminates the subjectivity of ICG angiography interpretation	-The model requires additional validation before widespread use
	Robotic assisted surgery with augmented reality	-Improved anatomical visualization, and intraoperative navigation	-Implementation and cost
**Postoperative**	Symptom monitoring	-Early detection of surgical site infection and flap compromise	-SSI model has only modest predictive accuracy -Liability and safety guidelines have yet to be established
	Patient education	-LLMs can deliver information in digestible language that suits the learning level of the patient	-Patient trust decreases without a clinician involved -Citation hallucination
	Postoperative esthetic Evaluation	-Neural networks offer an objective way to assess breast symmetry	-Limited empathy in delivering esthetic scores
